# Disease- and treatment-associated acquired glucocorticoid resistance

**DOI:** 10.1530/EC-18-0421

**Published:** 2018-10-11

**Authors:** Legh Wilkinson, Nicolette J D Verhoog, Ann Louw

**Affiliations:** Department of Biochemistry, Stellenbosch University, Stellenbosch, South Africa

**Keywords:** glucocorticoid receptor, glucocorticoid resistance, acquired resistance, biased ligands, GRα downregulation

## Abstract

The development of resistance to glucocorticoids (GCs) in therapeutic regimens poses a major threat. Generally, GC resistance is congenital or acquired over time as a result of disease progression, prolonged GC treatment or, in some cases, both. Essentially, disruptions in the function and/or pool of the glucocorticoid receptor α (GRα) underlie this resistance. Many studies have detailed how alterations in GRα function lead to diminished GC sensitivity; however, the current review highlights the wealth of data concerning reductions in the GRα pool, mediated by disease-associated and treatment-associated effects, which contribute to a significant decrease in GC sensitivity. Additionally, the current understanding of the molecular mechanisms involved in driving reductions in the GRα pool is discussed. After highlighting the importance of maintaining the level of the GRα pool to combat GC resistance, we present current strategies and argue that future strategies to prevent GC resistance should involve biased ligands with a predisposition for reduced GR dimerization, a strategy originally proposed as the SEMOGRAM–SEDIGRAM concept to reduce the side-effect profile of GCs.

## Introduction

Due to the interrelatedness of the stress and inflammatory responses, chronic persistent inflammation may be considered both a cause and a consequence of a prolonged disruption of the central HPA axis, a systemic signalling pathway of the stress response (1). This in turn, has many peripheral effects, such as an increase in circulating glucocorticoids (GCs) (2, 3).

Chronic stress or prolonged exogenous GC treatment also disrupts the central homeostatic nature of GC signalling, often resulting in various peripheral effects, one of which is the tissue-specific reductions in the glucocorticoid receptor α (GRα) functional pool. This reduction in the GRα functional pool may ultimately drive the development of acquired GC resistance and result in the progression of many psychological and pathological conditions.

Endogenous GCs, which are regulated by the HPA axis, are physiological mediators secreted in an ultradian or circadian manner (3) or in response to internal or external signals (2, 3, 4, 5, 6), such as infection, pain or stress, and function within the body to regulate inflammation and maintain internal homeostasis (2, 3, 6, 7). Exogenous GCs, designed to mimic the biological anti-inflammatory action of endogenous GCs, remain the mainstay therapeutic choice (7) for the treatment of chronic inflammation in various psychological and pathological conditions. GCs are currently one of the most widely prescribed drugs in the world with an estimated 1.2% of the population of the United States, using them (8). Although effective anti-inflammatory agents, it is believed that approximately 30% of all patients receiving treatment, experience a degree of GC insensitivity (9). Specifically, 4–10% of asthma patients (10), 30% of rheumatoid arthritis patients (10), almost all chronic obstructive pulmonary disease (COPD) (10) and sepsis patients (5) and 10–30% of untreated acute lymphoblastic leukaemia (ALL) patients (11) experience varying degrees of GC insensitivity.

This stochastic response to GCs within disease groups (10), is compounded by inter-individual variation in patient sensitivity, as well as tissue-specific intra-individual differences in GC responsiveness (1). Thus, research is now focussed on developing diagnostic tools for determining GC sensitivity prior to treatment, for the use in personalized therapeutic regimens (12), which will likely assist in limiting adverse side effects and restrict the development of further GC insensitivity.

This review begins by briefly describing the types of GC resistance and then discusses reductions in the GRα pool in various pathological and psychological conditions, in terms of acquired GC resistance. Primary focus is given to disease- or treatment-associated reductions in the GRα pool, which drive the development of GC insensitivity, followed by the molecular mechanisms involved in mediating these reductions. Furthermore, current methods to restore GRα protein expression and improve GC sensitivity are briefly detailed. Lastly, a potential role for the conformation of GRα in receptor turnover is proposed, and a strategy using conformationally biased ligands is advocated to combat acquired GC resistance.

## GC resistance

Following GC secretion into the bloodstream, GCs are transported to various tissues and cells and diffuse across the cell membrane where they bind and mediate their biological effects via their cognate receptor, the ligand-activated transcription factor, GRα (13). Upon ligand binding, the GRα undergoes a conformational change which allows for subsequent translocation to the nucleus (13). Here, the GC-bound GRα mediates the transrepression or transactivation of various GC-responsive genes (13, 14, 15).

Central to the ability of GCs to combat inflammation is the requirement for a significant amount of functional GRα through which they may mediate their effects (16, 17). There are a multitude of factors which can regulate the functional pool of GRα, either at the level of the functionality of the receptor and/or at the level of the GRα pool, thus ultimately contributing to GC resistance. In short, disruptions in GRα function (1, 7, 18) are known to modulate, not necessarily independently of one another, the subcellular localization, ligand binding and transactivation ability of the receptor, and are regulated by, among others, increases in additional GR isoforms (GRβ and GRγ) due to alternative splicing events, inactivating GRα mutations, the inflammatory cytokine profile of the cellular microenvironment and mutations/polymorphisms in the ERK pathway. However, rather than altered GRα function, the focal point of this review is reviewing the importance of the GRα pool, with regards to acquired GC resistance.

GC resistance is multi-faceted and has been extensively identified and studied in healthy and diseased states (9). Broadly speaking, GC resistance may be divided into two major groups: generalized (systemic/primary) or acquired (localized/secondary) GC resistance (1, 9). The generalized form of GC resistance falls beyond the scope of the current review, but for the interested reader is reviewed in several papers (1, 9, 15, 19). Essentially, these two groups of GC resistance are distinctively different in terms of the site of occurrence within a biological system, with acquired GC resistance often affecting specific tissues and/or cells while generalized GC resistance affects almost all tissues (1, 9). However, central to both types of GC resistance is perturbation of the GRα functional pool.

Acquired GC resistance is significantly more common in the general population and has been linked to a number of psychological and pathological conditions/diseases. An apt description for this form of GC resistance is a ‘consequence of a pathophysiological process’ (5) affecting specific tissues/cell types (9). Furthermore, the clinical use of GCs, although effective initially, may lead to the development of acquired GC resistance thus posing a significant challenge for the long-term treatment of these conditions (9).

GC-resistant patients often require higher GC doses for prolonged periods of time in order to efficiently combat chronic inflammation, which likely leads to adverse side effects and may aggravate GC insensitivity (16). Thus, it is of importance for practitioners to be able to evaluate the GC responsiveness, of individual patients, to permit personalized GC treatment to obtain an optimal therapeutic outcome (12). Acquired resistance is more difficult to diagnose than generalized resistance, which generally displays a ‘clinical picture’ of GC resistance (1). In terms of generalized GC resistance, no single, standardized method for determining patient sensitivity to GC treatment exists (12), however, a range of endocrine (1) (e.g. cortisol awakening rise/response (CAR) or the 24-h urinary-free cortisol (UFC)) and biochemical methods (9) (dexamethasone suppression test (DST) or the more recent Dex/CRH suppression test) are employed to determine generalized GC resistance. In contrast, patients with or developing acquired GC resistance are mostly asymptomatic, thus, a range of in depth biochemical diagnostic approaches (12, 20, 21) (e.g. BrdU incorporation lymphocyte steroid sensitivity assay (BLISS) and measuring the GC-responsive gene expression) are required to determine the GC responsiveness of specific tissues and/or cells. Although GC response can be determined, an increasing demand for more sensitive and specific tests remain, to avoid the unnecessary chronic GC use in treatment regimens (22).

## Reductions in the GRα pool and implications for acquired GC resistance

In many, but certainly not all, stress-related, psychological and pathological conditions, reductions in the GRα pool have been noted (9) (Table 1). These disease-associated reductions in the GRα pool often produce GC-resistant forms within disease groups, which are exceptionally challenging to manage clinically (9). In addition to the disease-associated reductions in the GRα pool, generally mediated via increased circulating endogenous GCs, GC treatment-associated reductions in the GRα pool are well documented (Table 2). It is often difficult to distinguish between disease- and treatment-associated GRα turnover because withholding GC treatment from patients would not be ethical. Moreover, the treatment-associated effects on the GRα pool often exacerbate those that are disease-associated (23), further contributing to the development of acquired GC resistance.

**Table 1:** Disease-associated reductions in the GRα pool.

Type of condition (general)	Broad category of disease condition	Species	Specific stress/condition/disease	Tissue/cells	GRα mRNA expression	GRα protein expression	Implications for GC sensitivity	References
Stress	Pre/post-natal stress	Humans	Pre-natal stressChildhood adversity/abuse leading to adult suicide	PBMCs^a^Hippocampus	Reduced	N.C^b^	N.D^c^	(24, 25, 26)
		Rodents	Early Life Stress (ELS) (i.e. maternal separation (MS) and preconception paternal stress (PPS))	Hippocampus, amygdala, limbic regions of brain dentate gyrus	Reduced	Reduced	Cognitive dysfunction, altered behavioural affects, increase in anxiety-like behaviour, anhedonia	(27, 28, 29, 30, 31, 32, 33, 34)
	Physical or psychological stress	Rodents	Restraint stress, psychological stress, forced swim stress (FSS), repeated social defeat (RSD), repetitive restraint stress (RSS), water-immersion and restraint stress (WIRS)	Hippocampus, amygdala, hypothalamus, cerebellum, splenic macrophages, splenocytes, peripheral leucocytes, oligodendrocytes of corpus callosum, prefrontal cortex, lung tissues	Reduced	Reduced	More susceptible to psychological disorders, asthma exacerbations, diminished GC sensitivity	(29, 37, 38, 39, 40, 41, 42, 43, 44, 45)
Psychological condition	Psychological conditions	Humans	Major depression (MD), schizophrenia, bipolar disorderPost-traumatic stress disorder (PTSD), general anxiety disorder (GAD)	Hippocampus, prefrontal-, temporal- and entorhinal cortex, PBMCs, lymphocytes	Reduced	N.D	Diminished GC sensitivityTreatment-resistant depression	(52, 53, 54, 55, 56, 57, 58, 59)
Pathological conditions	Autoimmune or inflammatory-linked conditions	Human	Atopic dermatitis (AD)	PBMCs	Reduced	N.D	GC resistant to topical treatment and systemic administration of potent corticosteroid	(61)
			Systemic lupus erythematosus (SLE)	PBMCs	Reduced	N.D	Diminished GC sensitivity	(62, 63, 64)
			Inflammatory bowel disease (IBD)	PBMCs	Reduced	N.C	Impaired GC response	(76)
			Adult immune thrombocytopenia (ITP)	PBMCs	Reduced	Reduced	GC-resistant ITP	(65)
			Asthma	PBMCs, cells from skin biopsies of patients	N.D	Reduced	GC-resistant asthma	(66, 67)
			Chronic obstructive pulmonary disease (COPD)	PBMCs, lymphocytes, lung tissue	Reduced	Reduced	GC-resistant COPD	(68, 69, 70, 71)
			Arthritis	Chondrocytes and lymphocytes	Reduced	Reduced	Steroid-resistant arthritis	(72, 73, 74)
		Rodents	Experimental encephalomyelitis (EAE)	T cells	Reduced	Reduced	GC-resistant apoptosis	(77)
	Cancer	Human	Acute lymphoblastic leukaemia (ALL)Multiple myeloma (MM)Small-cell lung cancer (SCLC), non-small-cell lung cancer (NSCLC), breast cancer	B-lineage leukaemia, T-ALL resistant, lymphoblasts, T-leukaemic, multiple myeloma, human carcinoma, lung adenocarcinoma cells, breast tissue	Reduced	Reduced	GC-resistant ALLGC-resistant MM and diminished GC sensitivity (transactivation and GC-mediated apoptosis)GC-resistant SCLC	(11, 79, 80, 81, 82, 83, 84, 85, 86, 87, 88, 89, 90, 91, 92, 93)
		Rodents	Liver cancer	HTC cells	Reduced	Reduced	Reduced sensitivity to Dex	(94)
	Infection and other conditions	Human	Sepsis	Neutrophils and T-cells	Reduced	Reduced	Diminished GC sensitivity	(95, 96)
			Idiopathic nephrotic syndrome (NS)	PBMCs	NC	Reduced	Steroid-resistant Nephrotic syndrome (SRNS)	(97)
			Keloid disease	Keloid tissue	Reduced	Reduced	Diminished GC sensitivity	(98)
		Rodents	Stroke	mouse brain capillary endothelial cells (cEND)	N.C	Reduced	Diminished GC sensitivity	(99)

^a^Peripheral blood mononuclear cells (PBMCs), ^b^No change in GRα expression (mRNA or protein) (N.C), ^c^Not detected (N.D).

**Table 2:** GC Treatment-associated reductions in the GRα pool.

Exogenous GC	*In vitro/ex vivo/in vivo*	Treatment conditions		Cells/tissues	GRα mRNA expression	GRα protein expression	Implications for GC sensitivity	References
		Concentration	Time					
Dex	*In vitro*^a^	Various Dex doses (10^−10^ to 10^−6^ M)	Generally up to 72 h with one study continuing treatment for up to 4 weeks and one for up to 2 years	Human IM-9 lymphocytes and rat pancreatic acinar (AR42J) cells Hepatoma tissue culture (HTC), HeLa, COS-1, cells NIH 3T3 cells, Chinese Hamster ovary-derived (CHO) cells, BWTG3 cells Mouse brain capillary endothelial (cEND) cells, U2-0S and A459, human respiratory epithelial cells (BEAS-2B) Normal human liver (HL7702) cells L6 muscle cells, fibroblast-like synoviocytes (FLS), RAW264.7 cells Peripheral blood mononuclear cells (PBMCs)	Reduced	Reduced	Most of the papers demonstrated diminished GC sensitivity	(99, 102, 103, 104, 105, 106, 107, 108, 109, 110, 111, 112, 116, 119)
	*Ex vivo*^b^ or *in vivo*^c^	5 μM, 20 μg or 1–5 mg/kg body weight	Up to 48 h, 3–28 days	Variety of mice and rat tissues (liver, kidney, lung and heart), culture mouse podocytes Rat hippocampal neurons Mice frontal cortex and hippocampus tissue Human lymphocytes	Reduced	Reduced	Most of the papers demonstrated diminished GC sensitivity	(60, 102, 107, 108, 113, 114, 115, 116)
Triamcinolone acetonide (TA)	*In vitro*	1 μM	Up to 96 h	L929 cells (a fibroblast-like cell line)	Reduced	Reduced	N.D^d^	(117)
Hydrocortisone	*In vivo*	Intraperitoneally 5 mg/100 g body weight	6 h	Liver tissue	N.D	Reduced	Altered GC sensitivity	(118)
Various prednisolone-based steroids	*In vitro*	10^−5^ M	0 to 24 h	HeLa	Reduced	N.D	N.D	(119)
	*In vivo*	120 mg/kgLow-dose and 1 × mega dose^e^; 0.01–0.3 mg/kg orally or 10–15 mg/kg i.v. pulse therapy^f^; 1 mg/kg body weight	10 daysDaily (oral) or 3 doses^e^; 4–6 weeks (i.v)	Liver tissueHuman blood monocytesLymphocyte subpopulationsPBMCs	Reduced	Reduced	Diminished GC sensitivity GC resistance based on clinical predictive factors for GC resistance (i.e. fundus depigmentation and chronic disease in VKH^g^)	(23, 100, 101, 120, 122)

^a^*In vitro*: GC treatment of transiently, stably transfected or endogenous GRα in tissue culture cells. ^b^*Ex vivo*: GC treatment of endogenous GRα in cells/tissues derived directly from animals in a tissue culture assay. ^c^*In vivo*: Subjects (rodents or patients) treated with GCs with cells/tissues retrieved and assayed (i.e. GC treatment does not occur in tissue culture). ^d^Not detected (N.D). ^e^See Berki *et al.* (122) for details. ^f^Intravenous therapy (i.v). ^g^Vogt–Koyanagi–Harada (VKH) disease (102).

### Disease-associated reductions in the GRα pool

There is a wealth of evidence associating stress, psychological and pathological conditions, with the development of an acquired GC resistance, through reductions in the GRα pool (Table 1).

Specifically, in terms of stress, the modulation of the GRα pool is fundamentally dependent on the duration of the stressor, the environment in which the stress occurs, and the individual’s sensitivity to stress (24, 25, 26, 27, 28, 29, 30, 31, 32, 33, 34, 35, 36, 37, 38, 39, 40, 41, 42, 43). Various stressors ranging from pre- or post-natal to physical and psychological stress, in a number of human and rodent studies, encompassing various different tissues and cells, result in significant reductions in the GRα mRNA and/or protein pool (Table 1). These reductions are generally, but not always (26), correlated with stress-induced increases in circulating endogenous GCs (24, 25). Whilst GC-mediated receptor turnover is thought to be an adaptive mechanism employed by the cell to protect against the damaging effects of unrelenting stress, this reduction in the GRα pool has implications in GC sensitivity, often leading to a blunted GC response (42). Jung *et al.* (38), supported by Quan *et al.* (43), noted reductions in the GRα mRNA, and protein pool following repeated social defeat in rodent models, and importantly correlated these reductions to a consequent diminished GC sensitivity. In addition to encouraging the development of GC resistance, certain chronic physical, psychological and/or pre- or post-natal stressors can also increase susceptibility to severe psychological or pathological conditions (44, 45). An example is a recent study by Han *et al.* (44) where stress-induced hypercortisolemia mediated a decrease in the GRα protein pool in the hypothalamus of mice, which subsequently increased their susceptibility to psychological disorders (e.g. depression).

In many psychological disorders, including depression and schizophrenia, a large cohort of patients, but not all (46, 47), display consistent biological findings (48, 49), namely an increase in inflammation and hyperactivity of the HPA, which drives hypercortisolemia, with consequences for the GRα pool in peripheral tissues (50). Whilst it must be noted that vast heterogeneity in GRα expression exists in patients with psychological conditions (48, 49, 50, 51, 52, 53, 54, 55, 56, 57, 58, 59), the current review focuses on conditions/disorders which have been explicitly linked to reductions in the GRα pool (Table 1). Specifically, a number of studies have demonstrated a reduction in the GRα mRNA pool in patients suffering from major depression (MD) (52, 53, 58), schizophrenia (58), bipolar disorder (58) and post-traumatic stress disorder (54, 56, 57, 59) in various tissues of the brain (e.g. the hippocampus and prefrontal cortex) as well as in peripheral blood mononuclear cells (52, 53, 58). Furthermore, in patients suffering from generalized anxiety disorder, a negative correlation was made between circulating GC concentrations and the GRα mRNA pool, which was subsequently shown to result in diminished GC sensitivity (55).

In terms of pathological conditions, it is difficult to tease apart whether modulations in the GRα pool are a pathological consequence of the disease, as in the case of many psychological disorders, or as a result of prolonged GC treatment, which many of these patients require (60). Nevertheless, this review highlights cases in which reductions in the GRα pool are noted in autoimmune or inflammatory-linked conditions, cancers and infection or other conditions, attempting to limit it to cases in which patients were not receiving treatment (Table 1).

In autoimmune and inflammatory-linked conditions, a significant correlation between disease-associated reductions in the GRα pool and GC resistance has been demonstrated for atopic dermatitis (AD) (61), systemic lupus erythematosus (SLE) (62, 63, 64), adult immune thrombocytopenia (65) (ITP), steroid-resistant Type II asthma (66, 67), chronic obstructive pulmonary disease (68, 69, 70, 71) and osteoarthritis in humans (72, 73, 74). However, it has been suggested that the level of the GRα pool is not the primary determinant for GC sensitivity in all inflammatory-linked conditions as in the resistant form of irritable bowel disease (75, 76) and rheumatoid arthritis (73), for example, a reduction in the GRα pool does not always correlate with GC resistance, nevertheless a partial role for the GRα pool likely exists. Furthermore, in a rodent model, T-cells obtained from mice with experimental autoimmune encephalomyelitis, have a reduced GRα mRNA pool, which was linked to diminished GC sensitivity, in terms of GC-resistant apoptosis (77).

GCs are a primary therapeutic choice in cancer for either their pro-apoptotic effects or their use as an adjuvant therapy, in combination with chemotherapeutic agents, to reduce symptoms such as inflammation, allergic reactions, pain and nausea, which may also be caused by the tumour itself (78). However, both the type of cancer cell as well as the level of the GRα pool of certain cancer cells are thought to play a significant role in mediating the response to GC treatment (78, 79, 80, 81, 82). It is fairly well documented that high GRα expression is associated with a good response to GC treatment in lung cancer; however, drastic reductions in the GRα pool, thought, in part, to be a pathological consequence of the tumorigenic process may lead to GC insensitivity. Specifically, a number of authors have detailed that a reduction in the GRα pool is negatively correlated to GC response (78, 79, 80, 81, 82). For example, in acute lymphoblastic leukaemia (ALL) (83, 84, 85, 86), multiple myeloma (MM) (87, 88, 89, 90, 91), lung cancer (i.e. small-cell lung cancer (SCLC) and non-small-cell lung cancer (NSCLC)) (78, 79, 80, 81, 82) and breast cancer (92, 93), reductions in the GRα pool, have been associated with treatment-resistant forms of these cancers and/or diminished GC sensitivity. Furthermore, Vanderbilt *et al.* (94) established that the GC response in a rat hepatoma cell line was modulated in accordance to the level of the GRα pool.

Apart from autoimmune and inflammatory-linked diseases and certain cancers, disease-associated reductions in the GRα pool have been documented in conditions such as sepsis (95, 96), nephrotic syndrome (NS) (97), keloid disease (98) and stroke (99). Although these reductions in receptor expression were generally negatively correlated to GC sensitivity, in sepsis, the association between the GRα pool and the GC response is, however, highly variable (5). In children with NS, the level of the GRα protein pool was assessed before exogenous GC treatment in two patient groups, namely the steroid-sensitive (SSNS) and the steroid-resistant (SRNS) groups (97). Patients from the SRNS group were reported to have reductions in the cellular GRα protein pool, which Hammad *et al.* (97) postulated may be one of the pathophysiological mechanisms of acquired GC resistance in these children. As with NS (97), patients with keloid disease may be separated into two groups, namely non-responders (nRPs) or responders (RPs) (98). Before receiving GC therapy, tissue isolated from keloid scars from nRPs displayed reductions in the GRα pool, both mRNA and protein, which was associated with decreased GC sensitivity following treatment (98). Lastly, in an *in vitro* model of hypoxia (used to mimic stroke events), endothelial cells isolated from mice brains, following O_2_/glucose deprivation had significant reductions in their GRα protein pool, relative to normoxic cells, which was proposed to be the cause of a decrease in subsequent GC sensitivity (99).

It is clear that chronic stress and certain psychological and pathological conditions drive disease-associated reductions in the GRα pool, often independently of exogenous GC treatment. More importantly, in many cases, these reductions in the GRα pool have been directly correlated to an increase in GC insensitivity and resistant forms of these diseases.

### GC treatment-associated reductions in the GRα pool

It is often difficult to discriminate between disease- and treatment-associated reductions in the GRα pool (60). However, some clinical studies have demonstrated treatment-associated reductions in the GRα pool independent of disease-associated reductions (100, 101). Using various *in vitro*, *in vivo* and *ex vivo* human and/or rodent models, a number of studies have demonstrated that exogenous GC treatment, e.g. with dexamethasone (Dex), results in significant dose- and time-dependent reductions in the GRα pool with implications for GC sensitivity (Table 2).

Specifically, *in vitro* Dex treatment led to time-dependent reductions in the GRα mRNA and/or protein pool, of between 50 and 90% (60, 99, 102, 103, 104, 105, 106, 107, 108, 109, 110, 111, 112, 113, 114, 115, 116). Interestingly, Dex treatment of HeLa cells conducted for 2 years, led to reductions in the GRα mRNA and protein pool to below detectable levels (103). Moreover, in most of these studies, where both the GR mRNA and protein pool was assessed, it would appear that the Dex-mediated reductions in the GRα protein pool were generally greater than that observed for the GRα mRNA pool. In a study by Bellingham *et al.* (112), the rapid Dex-mediated reduction in GRα protein expression was maintained even after 4 weeks, while GRα mRNA expression displayed a ‘biphasic pattern’, with an initial decrease followed by rise in receptor mRNA expression and a subsequent decline, which was attributed to ligand-induced transcriptional, post-transcriptional and translational regulation in mediating receptor mRNA expression, which was not reflected at the protein level (112). A number of studies using *ex vivo* and *in vivo* models mirror results of Dex-mediated reductions in the GRα mRNA and/or protein pool obtained in cell lines. In a variety of mouse tissues and rat liver tissue, prolonged treatment with Dex led to significant reductions in the GRα pool (60, 102, 107, 113, 114, 115, 116), which in some cases was associated with diminished GC sensitivity (102, 116).

Importantly, several *in vitro*, *ex vivo* and* in vivo* studies have demonstrated that GC sensitivity is compromised following prolonged Dex treatment, as a result of a significant reduction in the GRα pool (60, 99, 102, 103, 104, 105, 106, 107, 108, 109, 110, 111, 112, 113, 114, 115, 116), highlighting how long-term GC therapy contributes to the development of acquired GC resistance. In addition to Dex, Table 2 also summarizes the reductions in the GRα mRNA and/or protein pool mediated by other exogenous GCs (23, 101, 117, 118, 119, 120, 121, 122), such as hydrocortisone (118).

Taken together, both disease and/or exogenous GC treatment drive reductions in the GRα pool and development of acquired GC resistance, a major clinical challenge. With the burden of resistance to GC treatment mounting, it is of utmost importance to understand the molecular mechanisms involved in ligand-induced GRα turnover.

## Molecular mechanisms of GC-mediated reductions in GRα pool

To date, a number of GC-mediated molecular mechanisms employed by the cell have been identified to tightly regulate the GRα pool (Table 3).

**Table 3:** GC-mediated molecular mechanisms involved in reducing GRα expression.

Level of regulation	Molecular mechanism	Species	GRα mRNA expression	GRα protein expression	References
Epigenetic	DNA methylation of GRα gene • Rodents: exon 1_7_ • Humans: exon 1_F_, exon 1_C_, exon 1_B_, exon 1_H_, exon 1D	Rodent Human	Reduced Reduced	Reduced Reduced	(30, 32, 33, 42)(24, 25, 81, 92)
Transcriptional	GRα gene regulation via nGRE^a^ • Present in exon 6	Human	Reduced	N.D^b^	(107)
Post-transcriptional	miRNA • Rodents: miR-96, miR-101a, miR-142-3p, miR-433, miR-29b, miR-340-5p, miR-18 and miR-124a • Humans: miR-124, miR-130b and miR-142-3p	Rodent Human	Reduced Reduced	Reduced Reduced	(38, 40, 42, 127, 128, 129)(84, 89, 95, 130)
Post-translational	Phosphorylation • Rodents: ◦ Multiple mouse mutations(Ser212, Ser220 and Ser234) ◦ Hyper-phosphorylation at Ser412 • Humans: hyper-phosphorylation at Ser211, Ser226 and Ser404	Mouse Human	N.A^c^ N.A	Decreased Decreased	(136, 137)(135, 136)
	Ubiquitination • Rodents: K426 • Humans: K419 Proteasome degradation (i.e. use of proteasome inhibitors) • Rodents: MG132 or bortezomib (BZ) • Humans: MG132 or BZ	Mouse Human Mouse Human	N.A N.A N.A N.A	Decreased Decreased Decreased Decreased	(99, 104, 105, 139, 142)(105, 141, 143, 144, 145, 146)(99, 104, 105, 139, 142)(105, 141, 143, 144, 145, 146)
	Sumoylation • Specific site unknown	Human	N.A	Decreased	(152)

^a^Negative glucocorticoid response element (nGRE),^ b^Not detected (N.D), ^c^Not applicable (N.A) as effects exerted on GRα protein.

**Figure 1:**
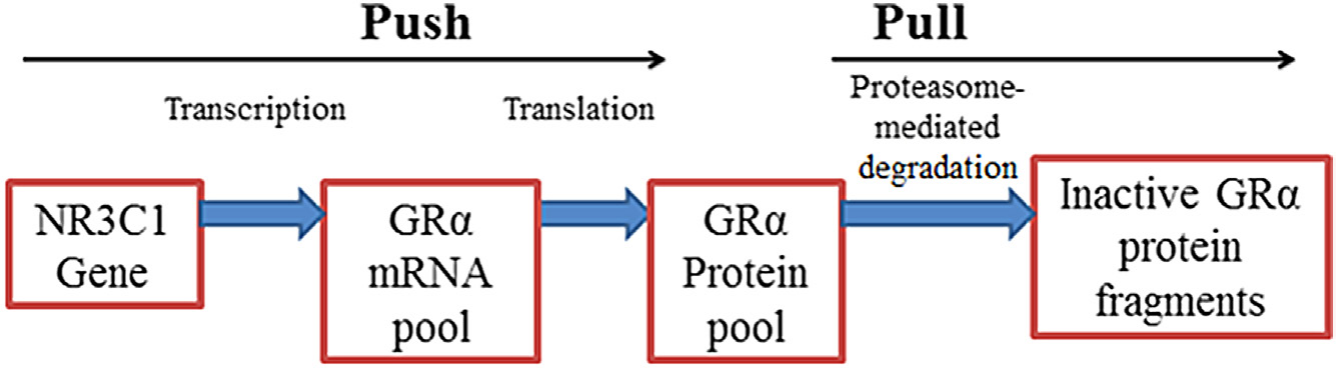
Regulation of the GRα protein pool described by a simple ‘push’ vs ‘pull’ mechanism.

The regulation of the GRα pool may be described using a simple ‘push’ vs ‘pull’ mechanism where, when in a dynamic state of equilibrium and unperturbed, the synthesis of GRα is roughly equivalent to receptor turnover and the level of the GRα pool remains constant (Fig. 1). The ‘push’ is governed by two processes namely transcription and translation while the ‘pull’ is defined by proteasomal degradation, specifically via the ubiquitin-proteasome pathway (UPS). One can assume that perturbations in the equilibrium state of GRα regulation will most likely result in alterations in the GRα pool. One of the ways in which the equilibrium of this dynamic state may be perturbed is via an increase in circulating GCs, either endogenous (i.e. disease-associated increases; Table 1) or exogenous (due to prolonged treatment; Table 2), which subsequently induces GC-mediated GRα turnover.

GC-mediated regulation of the GRα pool is complex and involves multiple layers of epigenetic, transcriptional, post-transcriptional and post-translational regulation (9, 15). At each level of regulation, the molecular mechanisms function in a highly specific manner to stabilize or destabilize the GRα, which contributes to the complexity of the finely tuned GC/GRα signalling pathway, with receptor destabilization potentially advancing acquired GC resistance. This review focuses specifically on the molecular mechanisms, which function to reduce the GRα mRNA and protein pool in a ligand-dependent manner, however, ligand-independent regulation has been described (1, 9, 15).

### GRα mRNA regulation

#### Epigenetic regulation

DNA methylation of the GRα (NCR31) promoter (123) has been identified as one of the major mechanisms involved in disease-associated acquired GC resistance across species (24, 25, 30, 32, 33, 42, 55, 81, 92) and has been positively correlated with an increase in circulating GCs (42). GC-mediated increases in DNA methylation of the GRα promoter generally, but not always (47), lead to a reduction in the GRα mRNA pool and possibly a corresponding reduction in the GRα protein pool (Table 3).

A specific exonic sequence in the rat GRα gene has been identified as a region that undergoes substantial DNA methylation following stressful events (30, 32, 42). Specifically, increased DNA methylation at the exon 1_7_ promoter, within the GRα promoter, was shown to mediate a reduction in the GRα mRNA pool (30, 32, 42), with Mifsud *et al.* (42), demonstrating up to a 75% reduction in the GRα mRNA pool in dentate gyrus neurons of male Wistar rats. In mice, methylation of the same exon 1_7_ promoter led to a significant reduction in the GRα protein pool (33). Additionally, human studies have demonstrated that DNA methylation of the GRα gene, specifically at exon1_F_, exon 1_D_, exon 1_B_, exon 1_H_ and exon 1_C_, resulted in reductions in the GRα mRNA pool (24, 25, 55, 81, 92). DNA methylation of the exon 1_F_ promoter led to reductions in the GRα mRNA pool in tissues/cells from victims with a history of abuse (25) and patients with generalized anxiety disorder (55), with the latter being correlated to diminished GC sensitivity (55). Similarly, for exon 1_B_, exon 1_C_ and exon 1_H_, an increase in the methylation status at these sites was associated with a decrease in the GRα mRNA pool, in breast cancer tissue (92) and the hippocampi of suicide completers (24). Furthermore, Kay *et al.* (81) showed that a 6% increase in GRα methylation resulted in a reduction in the receptor protein pool by up to 50%, in human small-cell lung cancer cells. Collectively, these studies highlight a role for DNA methylation in GC-mediated reductions in the GRα pool and demonstrate that this epigenetic mechanism is likely to contribute to the development of acquired GC resistance.

#### Transcriptional regulation

The GRα promoter has a negative glucocorticoid response element (nGRE) (107, 124). GC-mediated inhibition of transcription initiation of the GRα gene was shown to be the primary mechanism for up to a 90% reduction in the nascent GRα mRNA pool (107). Specifically occurring through a long-range interaction between the GC-bound GRα, at a nGRE present in exon 6, and a NCOR1 repression complex, which is assembled at the transcription start site of the gene (107). The ability of the GC-bound GRα to regulate its own transcription was neither species nor tissue specific (107). Whilst Ramamoorthy *et al.* (107) convincingly demonstrated that the GC-mediated auto-regulatory loop to repress the GRα gene occurs via an nGRE in the GRα gene promoter; it appears to be the only study to do so.

#### Post-transcriptional regulation

Unlike transcriptional regulation of the GRα gene that modulates nascent receptor mRNA expression, post-transcriptional regulation involves the destabilization of mature receptor mRNA via the presence of adenylate uridylate (AU)-rich elements present in the 3′-untranslated region (UTR) of the GRα mRNA transcript, which may ultimately affect receptor protein expression, presenting another level of regulation for fine-tuning GRα expression (125). One of the ways in which this can occur is through the regulatory role of miRNAs, which bind to 3′-UTR of GR mRNA (22). These miRNAs are a family of small non-coding RNAs, which primarily prevent efficient translation of mRNA transcripts but can also induce degradation of these transcripts (126).

The ability of miRNAs to regulate the GRα mRNA pool has been shown to be GC mediated and has been implicated in acquired GC resistance (Table 3). Vandevyver *et al.* (15) reviews most, but not all (38), of the miRNA target sites in the GRα mRNA transcript; however, the current review will focus only on miRNAs which reduce the GRα pool. Four miRNAs, namely miR-96, miR-101a, miR-142-3p and miR-433, drive reductions in the GRα mRNA pool by up to 40% in mice (127). Additionally, social stress in mice (38) and acute stress in rats (42), resulted in an increase in miR-29b and miR-340-5p and miR‐124a expression, respectively, which was associated with a significant reduction in the GRα mRNA pool. Reductions of the GRα protein pool in rats not necessarily reflected at the mRNA level have also been noted as a result of an increase in miR-18 (128, 129) and miR-124a (40). In humans, a reduction in the GRα pool (both mRNA and protein) was noted following a GC-mediated increase in miR-124, in ALL cells (130) and in T-cells of sepsis patients (95). Moreover, Tessel *et al.* (89) demonstrated that overexpression of miR-130b mediated a reduction in the GRα protein pool in human MM cell lines; however, knockdown of this miR-130b did not alter GRα protein levels and whilst experiments were conducted in the presence of Dex, it is not clear whether GC’s directly mediated the expression of miR-130b (89). Moreover, an increase in miR-142-3p expression and consequent decrease in the GRα protein pool has been noted in GC-resistant ALL patients (84). Unfortunately, in many of these studies, it is unclear whether up to 80% increase in miRNA expression (38) is directly mediated via an increase in circulating GCs; however, from other studies, one could postulate that a positive correlation between the two exists.

### GRα protein regulation

#### Post-translational regulation

Additionally, the GRα protein is also subjected to GC-mediated regulation in the form of post-translational modifications (PTMs). The nature and degree of these PTMs modulates both GRα function and pool, impacting GC responsiveness in selective tissues, and in some cases, contributes to an acquired GC resistance (15). In this review, we focus on GC-mediated PTMs, which drive reductions in the GRα pool via the proteasome. The effects of PTMs on GRα function are reviewed in several papers (7, 13, 15, 131, 132, 133, 134).

**Figure 2:**
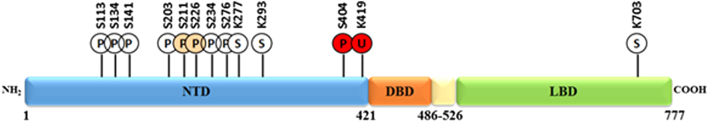
Post-translational modification sites of human GRα with focus on phosphorylation, ubiquitination and sumoylation. The human GRα protein consists of 777 amino acids and undergoes PTMS at numerous sites. Moreover, many of these PTM sites are contained within the N-terminal domain (NTD) (amino acids 1 to 421) of the receptor, with two present in close proximity to the DNA-binding domain (DBD) (amino acids 421 to 486). Specifically, phosphorylation (P) occurs at serine (e.g. S211, S226 and S404) residues, whilst ubiquitination (U) and sumoylation (S) occurs at lysine residues (i.e. K419 and K277, K293 and K703, respectively). Unlike the others, the K703 sumoylation site occurs within the ligand-binding domain (LBD) of the receptor (amino acids 526 to 777). Moreover, PTMs at these sites are known to modulate GRα function (white) or protein expression (red) and in some cases affect both receptor function and protein expression (pink).

For GRα, the most widely studied and first PTM identified was phosphorylation (15). Since the initial discovery, additional GRα phosphorylation sites have been identified (Fig. 2). Basal GRα phosphorylation may occur in a ligand-independent manner (135, 136), however, hyper-phosphorylation at several of these sites is GC-mediated (135, 136) and modulates GRα function as well as the receptor pool (15, 135, 136). Moreover, various kinases (e.g. p38, ERK, JNK, CDKs and GSK3β (136)) responsible for the phosphorylation of these sites have been described (15).

Webster *et al.* (137) demonstrated that multiple point mutations (i.e. at S212, S220 and S234) in the mouse GRα, which correlate to S203, S211 and S226 of the human GRα (15), respectively, restricted GC-mediated GRα protein turnover. GC-mediated hyper-phosphorylation of the human GRα at S211, S226 (135) and S404 (136) (or Ser412 in mice (136)) led to reductions in the GRα protein pool. Moreover, inhibiting the GC-mediated hyper-phosphorylation at S404, through the use of a mutant or a kinase inhibitor, resulted in a significant increase in GRα protein stability (136). To our knowledge, these are the only sites (135, 136, 137), which directly demonstrate the ability of GC-mediated phosphorylation of the human GRα (135, 136) and the mouse GRα (137) to affect the GRα pool.

It was postulated, but not demonstrated experimentally, that, apart from the inability to be phosphorylated, the phospho-deficient GRα mutants (137, 138), could not be ubiquitinated. Protein ubiquitination is preceded by phosphorylation and is a fundamental requirement for protein degradation via the proteasome; however, GRα ubiquitination is not well documented with only a handful of papers specifically demonstrating GC-mediated GRα ubiquitination (99, 104, 105, 139, 140, 141, 142, 143, 144, 145, 146). Moreover, the idea that ubiquitination of GRα increases following GC treatment seems to be controversial, with one paper demonstrating a Dex-mediated increase in GRα ubiquitination (140) while others noted a Dex-induced reduction in GRα ubiquitination in the presence of a proteasome inhibitor (105, 142). It seems necessary for further research to be conducted in this specific area of GC/GRα signalling. To date, only a single ubiquitination site for GRα that occurs within the PEST degradation motif at Lys426 in mice and Lys419 in humans has been identified, with mutations at these sites restoring the GRα protein pool, by restricting GRα turnover via the proteasome (104, 105). Nevertheless, several studies have through the use of proteasome inhibitors, definitively implicated the ubiquitin-proteasome system (UPS) in the control GRα degradation rates, ultimately contributing to the stringent regulation of the GRα protein pool (99, 102, 104, 105, 139, 142, 145, 147).

Similarly to ubiquitination, sumoylation is a dynamic, reversible process, which involves a multi-step, enzyme-catalysed reaction to mediate the covalent attachment of the SUMO protein (e.g. SUMO-1, SUMO-2/3) to the protein of interest (148). Sumoylation of the GRα is known to modulate GRα function (131, 149, 150, 151, 152) and, less frequently, promote reductions in the GRα pool (152). Specifically, Le Drean *et al.* (152) demonstrated that overexpression of SUMO-1 aids Dex-mediated receptor downregulation; however, this paper is the only paper to describe the potential of sumoylation to regulate GRα protein expression.

#### Enzymes of the UPS that mediate GRα protein turnover

**Figure 3:**
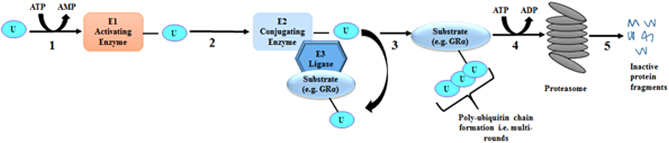
The ubiquitination of a substrate requires multiple rounds of a multi-step enzymatic process before being targeted to the proteasome. 1. Ubiquitin (U) is activated by an activating enzyme (E1) in an energy (ATP)-dependent manner. 2. The activated U molecule is then transferred to E2, a conjugating enzyme. 3. E3 binds the substrate and the E2 and the transfer of the activated U molecule from E2 to the substrate occurs. 4. This is repeated, until a poly-ubiquitinated chain is formed and the ubiquitinated substrate is then actively (i.e. ATP-dependent) delivered to the proteasome. 5. The catalytically active proteasome recognizes and degrades the substrate to produce inactive protein fragments.

Proteasomal degradation of a substrate (i.e. GRα) requires rounds of ubiquitination, mediated by various enzymes of the UPS (Fig. 3) to form a poly-ubiquitin chain, which the proteasome recognizes, resulting in degradation. There are number of UPS enzymes and additional co-regulators (153, 154, 155, 156, 157, 158, 159), which interact with the GRα protein (Fig. 3), in a GC-dependent or independent manner, as regulators of the GRα pool and function. The co-regulator/GRα interactions, which mediate reductions in the GRα pool via the ubiquitin-dependent proteasomal degradation pathway (104, 105) have implications in GC sensitivity and is the primary focus of this section.

The binding of two enzymes associated with the UPS, namely the inactive E2 conjugating enzyme, tumour susceptibility gene 101 (TSG101) (160) or the E3 ligase, carboxy-terminus of heat shock protein 70-interacting protein (CHIP) (161), to the GRα protein does not require prior ligand binding (Table 4). Moreover, whilst binding of CHIP to GRα is unaffected by GC treatment (139), the formation of the TSG101/GRα complex only occurs in the absence of ligand binding (162). Specifically, TSG101, which like the unliganded GRα is located in the cytoplasm, binds to the N-terminal region of the hypo-phosphorylated unliganded receptor and prevents protein turnover of the unliganded GRα by acting as a dominant negative regulator of ubiquitination due to its catalytically inactive characteristic (162, 163). Knockdown experiments in which TSG101 was targeted demonstrated a decrease in the stability of the hypo-phosphorylated form of GRα, thus suggesting a role for TSG101 in protecting the unliganded GRα from receptor turnover (162). A mutant GRα receptor (S203A/S211A), incapable of undergoing even basal phosphorylation showed enhanced interaction with TSG101 (162), indicating that the association of GRα with TSG101 is dependent on the GRα phosphorylation status. Unlike TSG101, CHIP interactions with GRα seems to be phosphorylation and ligand independent, however, it appears to be a major regulator of unliganded receptor turnover (164) and its presence in the cell is vital for basal GRα protein turnover (139). Overexpression of CHIP in HT22 cells, where steady-state receptor levels were unaffected by prolonged hormone treatment, is able to restore GC-mediated GRα protein turnover, confirming a role for this E3 ligase in reducing the GRα pool (139).

**Table 4:** Enzymes of the UPS that mediate GRα protein turnover.

Enzyme	Type of UPS enzyme	Interactions with GRα depend on				Role in GRα turnover	References
		Ligand-binding status		Phosphorylation status			
		Unliganded	Liganded	Hypo	Hyper		
TSG101	inactive E2 conjugating enzyme	Yes	No	Yes	No	Protects unliganded GRα from turnover	(162)
UbcH7	E2 conjugating enzyme	No	Yes	No	Yes	GC-mediated turnover	(169)
CHIP	E3 ligase	Yes	Yes	Yes	Yes	GC-mediated and basal turnover	(139)
FBXW7α	E3 ligase	No	Yes	No	Yes; at S404	GC-mediated turnover	(136, 167)
Mdm2/Hdm2	E3 ligase	Yes, but requires p53	Yes, but requires p53	Yes	Yes	GC-mediated turnover	(140,144, 155)

Binding of F-box/WD repeat-containing protein 7 (FBXW7α), an E3 ligase, to its substrate, requires substrate phosphorylation at a CDC4 phosphodegron motif (165) to mediate phosphorylation-dependent ubiquitination and subsequent proteasomal degradation (166). Specifically, FBXW7α binding to GRα is primarily dependent on GSK3β-mediated phosphorylation at S404 (136), which then targets it for proteasomal degradation (167). Malyukova *et al.* (167) demonstrated that a GRα phosphorylation mutant (S404A) was incapable of GC-mediated ubiquitination, which partially restricted its degradation via the proteasome. In addition, inactivation of FBXW7α, via mutations, restricted GRα protein turnover (167). From this evidence, it is clear that FBXW7α activity and expression has implications for GC sensitivity by regulating GC-mediated reductions in the GRα pool.

Ubiquitin-conjugating enzyme (UbcH7), an E2-conjugating enzyme, is a known co-regulator of steroid hormone receptors (168), including the GRα. It has been shown to modulate the function and level of the GRα pool, by targeting the receptor for degradation in response to GCs (169). Immunofluorescence studies have elucidated that UbcH7 is predominantly co-localized with GC-bound GRα in the cell’s nucleus, however, cytoplasmic UbcH7 was also observed (169). Overexpression of a dominant negative form of UbcH7 preserved the GRα pool through increasing the stability of the receptor and restricting GC-mediated GRα turnover, thus confirming UbcH7 as a key regulator of the GRα pool and supporting a role for UbcH7 in mediating GC sensitivity (169).

Lastly, another UPS enzyme involved in the regulation of the GRα pool is the E3 ligase, murine double minute 2 (i.e. Mdm2 (144) or Hdm2, the human homologue (170)). Unlike the other enzymes, Mdm2 relies on the presence of p53 to form a trimeric complex with GRα to mediate receptor proteasomal turnover, both in the presence and absence of GCs (155). Dex treatment of human umbilical endothelial cells enhanced GC-mediated ubiquitination of GRα in the presence of all three proteins (i.e. GRα, p53 and Hdm2) (140). Furthermore, disruption of the interaction of p53 with Hdm2 prevented Dex-induced ubiquitination of GRα (140). Interestingly, both the presence of Mdm2 and p53 where required for oestrogen-mediated GRα protein turnover, via the proteasomal degradation pathway (144).

## Strategies to restore the GRα pool for improved GC sensitivity

It is clear that reductions in the GRα pool, whether disease-associated (Table 1), treatment-associated (Table 2), or both, contribute to the development of acquired GC resistance. With the increasing incidence of severe stress, psychological and pathological conditions, in combination with the looming threat of acquired GC resistance, a dire need exists for the development of novel GC therapeutics to combat chronic inflammation, without eliciting GC resistance.

### Current strategies

In recent years, as discussed, a number of molecular mechanisms involved in GRα turnover have been uncovered and these have been explored and in some cases utilized in a clinical setting (40, 99, 102, 145).

For example, proteasome inhibitors, such as MG132 (104, 105), used in tissue culture cells, and bortezomib (BZ), used clinically (145) may prevent GC-induced GRα downregulation. Moreover, the repurposing of BZ, a Food and Drug Administration (FDA)-approved therapeutic (146), has been shown to restore GC sensitivity by preventing receptor turnover (99, 145). Specifically, in a model of hypoxic blood–brain barrier damage, O_2_/glucose deprivation led to an approximate 80% reduction in the GRα protein pool, with BZ treatment restoring the receptor pool to 90% (in the absence of Dex) or 50% (in the presence of Dex) (99). Importantly, this restoration in the GRα pool was associated with increased GC sensitivity (99). Additionally, Lesovaya *et al.* (145) demonstrated the ability of BZ to increase the anticancer activities of GCs, by maintaining the GRα pool through proteasomal inhibition. Although proteasomal inhibition (99, 104, 105, 145) seems promising for restoring GC sensitivity, chronic inhibition of such a vital system for finely tuning the levels of numerous proteins (171) could be risky.

Other compounds, such as Yokukansan (YKS) (a Japanese herbal medicine for the treatment of psychiatric and psychological symptoms (172, 173)) and Ginsenoside Rh1 (102) (a major active compound in Ginseng (174)) have also been shown to exert a protective effect against GC-mediated GRα turnover. Specifically, YKS counteracted by approximately 20% a stress-induced reduction in the GRα protein pool in mice (40) through a molecular mechanism that reduced (by almost 50%) the expression of miR-124, which targets GRα mRNA. Combinatorial treatment of Ginsenoside Rh1, with Dex, restricted reductions in the GRα pool, thus potentiating Dex’s anti-inflammatory potential, specifically in prolonged treatments (102). Whilst the ability of Ginsenoside Rh1 was found to require mRNA transcription and new protein synthesis (102), suggesting its ability to transcriptionally and post-transcriptionally regulate the GRα pool, the exact mechanism, remains to be elucidated.

### Future strategies

To date, current strategies to restore GRα levels for improved GC sensitivity have been based on combinatorial treatments and have not focussed on GRα ligands biased towards preventing a decrease in the receptor pool. Biased ligands, defined by Luttrell *et al.* (175) as ‘novel pharmacologic entities that possess the unique ability to qualitatively change receptor signalling’, may display an increased efficacy and/or a defined functional selectivity (14, 134), which could be harnessed to improve the therapeutic index of GCs. Additionally, Luttrell *et al.* (175) makes a strong case that the biological responses that arise from the interaction of a ligand with its cognate receptor are all encoded at that single point of contact with a distinct conformational change in the receptor being the initial consequence of ligand binding. Thus, conformationally biased ligands drive the conformational equilibrium towards a particular state, resulting in differential biological responses downstream.

**Figure 4:**
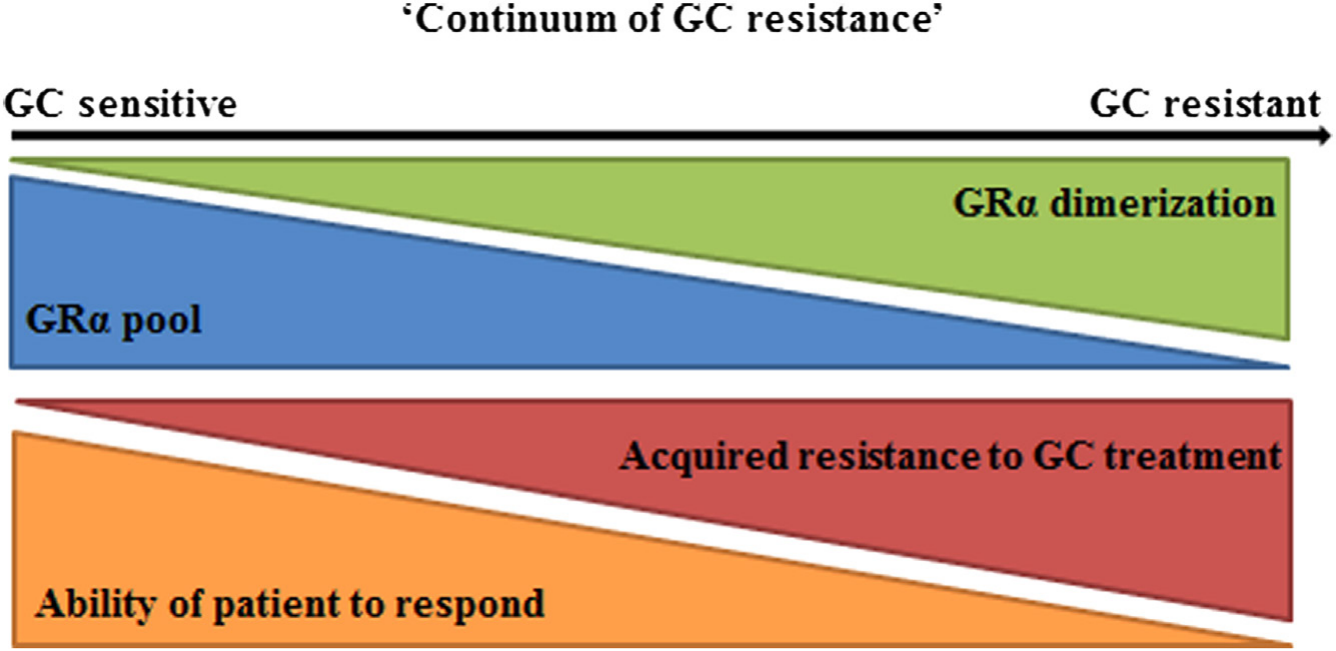
A ‘continuum of GC resistance’. As GRα dimerization increases, so increased ligand-induced receptor turnover of the GRα pool, both at the mRNA and protein level, occurs. These significant reductions in receptor turnover, in many cases, drive the development of an acquired resistance to treatment and so the ability of a patient to respond to treatment diminishes.

Recently, De Bosscher *et al.* (176, 177) developed the SEMOGRAM–SEDIGRAM strategy, which is essentially based on conformationally biased ligands that induce either monomers (SEMOGRAMs) or dimers (SEDIGRAMs) of the GR for use as selective therapeutics in chronic or acute inflammation, respectively. Whilst De Bosscher *et al.* (176, 177) address selectively modulating the dimerization state of GRα in terms of the anti-inflammatory effects of GC signalling vs their adverse side effects, the ligand-selective effects of GRα conformation on receptor turnover, with implications in acquired GC resistance, are not addressed. We now suggest that co-opting the SEMOGRAM–SEDIGRAM strategy for acquired GC resistance could be fruitful and propose the idea of a ‘continuum of resistance’ (Fig. 4), where encouraging GRα dimerization though the use of SEDIGRAMs, may not only have negative implications in terms of the generation of adverse side effects (178, 179), but may also drive reductions in the GRα pool, which encourages a decrease in GC sensitivity. In contrast, the use of SEMOGRAMs, which abrogate GR dimerization, may result in reduced side effects and prevent acquired resistance while maintaining an adequate anti-inflammatory potential, a therapeutic regimen more suited to chronic use.

In support of this, a wealth of pharmacological evidence supports the biased ligand behaviour of the SEMOGRAM, Compound A (CpdA) (180). The biased ligand behaviour of CpdA arises from its ability to abrogate GRα dimerization (181, 182), which favours transrepression of pro-inflammatory genes, which contributes to its potent immunosuppressive effects, over transactivation, generally associated with negative side effects and has proved effective in combatting inflammation in a number of *in vivo* models (116, 176, 183, 184, 185, 186, 187) without resulting in adverse side effects (116, 184, 186, 188, 189). Furthermore, CpdA does not result in ligand-induced GRα turnover (106, 116, 135), an ability that may be related to its ability to abrogate GR dimerization, and as such may be considered a biased ligand able to prevent acquired resistance. In fact, recent work from our own laboratory demonstrates that dimerization impairment, either through the use of CpdA or the dimerization deficient GR mutant (GRdim), restricts GRα turnover via the proteasome through a molecular mechanism involving a substantial reduction in hyper-phosphorylation at Ser404 and the interaction of GR with the E3 ligase, FBXW7α (190).

Throughout this review reductions in the GRα pool mediated by classical GCs, such as Dex, of anywhere between 10 and 90% have been detailed, which promote GC resistance (Tables 1 and 2). Importantly, these GCs are known to induce GRα dimerization of the GRα (181, 182, 191), prior to eliciting a biological response and subsequently driving receptor turnover, and may thus be termed dimerization promoting GCs or SEDIGRAMs. On the other hand, CpdA, which displays dimerization abrogating potential and is thus a SEMOGRAM, does not induce GRα turnover (106, 116, 135, 190) while maintaining its immunosuppressive capabilities even during prolonged treatment regimens (106, 116). We believe, this begs the question of whether the dimerization state of the GRα is likely to influence development of an acquired resistance to treatment, in prolonged GC regimens.

Caution should, however, be exercised in over­enthusiastically embracing GR ligands conformationally biased towards loss of dimerization for prevention of acquired GC resistance as our understanding of the implications of GR dimerization in GC signalling is currently limited. Accordingly, a more prudent approach may be the development of biased ligands positioned along the continuum of GC resistance (Fig. 4) rather than at the extremes of the monomer/dimer dichotomy. Nonetheless, in addition to the current strategy of combinatorial use of compounds (99, 102, 145) that may restrict receptor turnover, we believe that disrupting dimerization through biased ligands, in a tissue-specific manner, may be a fruitful future strategy for developing tailored treatments to counteract the development of acquired GC resistance in a number of disease states. Moreover, an in-depth characterization of the dimerization capabilities (192) of GRα mutants (51), associated with generalized GC resistance, may provide more insight into generalized GC resistance and assist in the treatment of these rare, pathological conditions.

## Conclusions

To conclude, acquired GC resistance, due to reductions in the GRα pool, is an ever-increasing therapeutic challenge for patients requiring chronic treatment and occurs ubiquitously throughout a number of psychological and pathological conditions. In recent years, a number of the molecular mechanisms which underpin these GC-mediated reductions in the GRα pool have been elucidated, with attempts to counteract GC-mediated receptor turnover being made through combinatorial treatment of GCs with other compounds, which disrupt transcriptional, post-transcriptional and post-translational GRα regulation. Whilst in some cases, these strategies have proved fruitful, they are not without limitations. Thus, we believe the strategy of using conformationally biased ligands, specifically the SEMOGRAM–SEDIGRAM strategy, which underscores the importance of GRα conformation, with particular reference to the receptor’s dimerization state, requires investigation and offers a novel perspective from which to approach the rational design of drugs that limit GC resistance.

## Declaration of interest

The authors declare that there is no conflict of interest that could be perceived as prejudicing the impartiality of this review.

## Funding

This work was supported by the National Research Foundation, South Africa (grant CPRR14072479679 to A L and PhD bursary to L W). Any opinion, findings and conclusions or recommendations expressed in this material are those of the author(s) and therefore the NRF do not accept any liability in regard thereto.

## Author contribution statement

A L, N J D V and L W participated in drafting the manuscript, revising its intellectual content and approved the final version of the submitted manuscript.
